# Toxicokinetics of a Single Oral Dose of OTA on Dezhou Male Donkeys

**DOI:** 10.3390/toxins15020088

**Published:** 2023-01-18

**Authors:** Ruifen Kang, Honglei Qu, Yanxin Guo, Mengjie Zhang, Tianze Fu, Shimeng Huang, Lihong Zhao, Jianyun Zhang, Cheng Ji, Qiugang Ma

**Affiliations:** 1 State Key Laboratory of Animal Nutrition, College of Animal Science and Technology, China Agricultural University, Beijing 100193, China; 2 National Engineering Research Center for Gelatin-Based Traditional Chinese Medicine, Dong-E-E-Jiao Co., Ltd., Liaocheng 252201, China

**Keywords:** toxicokinetic, toxicity, mycotoxins, ochratoxin, donkey

## Abstract

Ochratoxin (OTA) is widely present in a wide range of foods and feeds, causing adverse effects on animals and humans. This study aims to explore the toxicokinetics of OTA-contaminated materials on the Dezhou male donkey. Donkeys received a single orally dose of 2500 μg OTA/kg BW, obtained from *Aspergillus ochraceus* culture material. The concentrations of OTA in plasma collected at 0, 5, 10, 15, 20, 30, 45 min, and at 1, 1.5, 2, 3, 6, 9, 12, 24, 48, 72, 96 and 120 h were detected by HPLC. OTA eliminated in urine and feces were quantified at 6-h intervals up to 24 h and then at 4-h intervals up to 120 h. The results suggested that the maximum concentration of OTA in plasma was observed at 12 h after administration, with a mean value of 10.34 μg/mL. The total excretion in both urine and feces was about 10% of the intake until 120 h.

## 1. Introduction

Ochratoxin A (OTA) is among the secondary metabolites produced by *Aspergillus species* and *Penicillium species* [1], which are widely presented in crops, fruits, nuts and meat products [2], causing diverse toxicities in the host, including immunotoxicity [3,4], nephrotoxicity [5,6], hepatotoxicity [7], and carcinogenicity [8]. OTA enters the organism and tends to accumulate mainly in the liver and kidney, which are its key target organs in exerting toxic effects [9,10,11]. The toxicokinetics of OTA have been studied in various animals, including pigs, chickens, rabbits, rats, mice, fishes, quails, monkeys, turkeys and ducks, by oral or injection, most of them using purified toxin or culture material containing OTA [12,13,14,15,16,17]. The absorption rate of OTA is high, especially in pigs (up to 65.7%), and the half-life is long, ranging from 4.1–88.8 h in pigs, chickens and rabbits [12]. The rapid absorption and slow elimination of OTA result in its accumulation in animals and animal products for a long time, further passing to humans through the food chain, endangering human health.

Equine species have high economic value, due to their suitability as sports and competition animals, companion animals, and service domestic animals, and they also provide meat and donkey-hide gelatin for humans. However, there is little information to be found about the toxic effects of OTA toxin on equine species. Thus, the present study was designed to explore the toxicokinetics of OTA in donkeys after receiving a single oral dose of OTA containing *Aspergillus ochraceus* culture material.

## 2. Results

### 2.1. Validation Parameters

**Figure 1:**
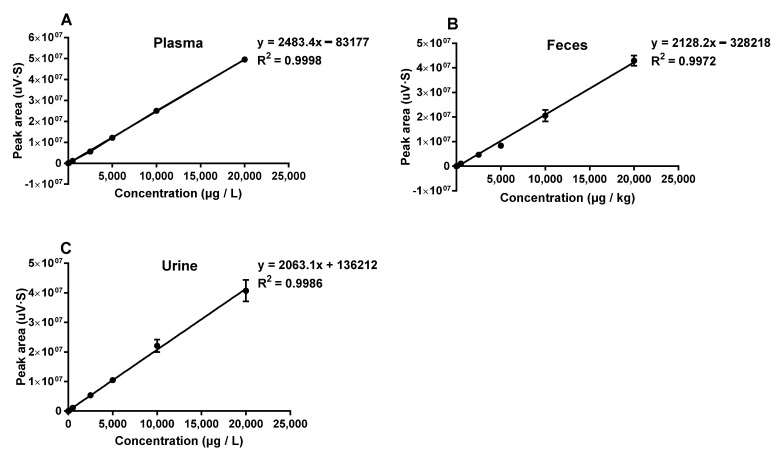
Calibration curves for spiked samples (1, 5, 10, 50, 100, 500, 2500, 5000, 10,000, 20,000 (μg/L)/(μg/kg) of (**A**) plasma, (**B**) feces and (**C**) urine, *n* = 3.

Calibration curves showed a linear trend in the range of 1–20,000 μg/L (Figure 1), with a coefficient of determination (R^2^) of 0.9998, 0.9986 and 0.9972 for OTA of plasma, urine and feces, respectively. For OTA in plasma, urine and feces, the limit of detection (LOD) was 0.2 μg/L and the limit of quantification (LOQ) was 1.0 μg/L (Table 1). Recovery was assessed at five levels in plasma, urine and fecal samples with mean values of 89.50, 86.72 and 83.19% for OTA, respectively (Table 2).

### 2.2. Toxicokinetic Parameters of OTA in Donkey Plasma

After a single oral dose of OTA (2500 μg/kg BW) in donkeys [12], the toxin was absorbed into the blood circulation. OTA in collected plasma samples was detected. As shown in Table 3, the concentration of OTA in plasma reached a peak concentration of (10.34 ± 2.05) μg/mL at 12 h. The elimination half-life was 24.52 ± 2.48 h, and the area under plasma concentration-time curve was 656.20 ± 99.49 μg·mL^−1^·h.

### 2.3. Plasma Concentration of OTA

**Figure 2:**
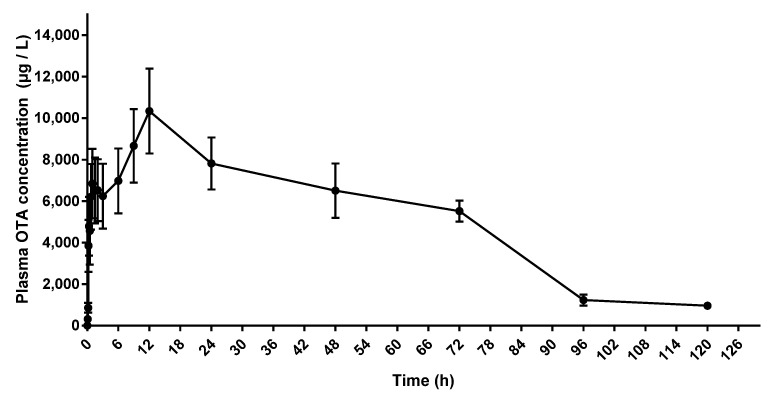
Plot of plasma mean concentration of OTA vs. time (0, 5, 10, 15, 20, 30, 45 min, and 1, 1.5, 2, 3, 6, 9, 12, 24, 48, 72, 96 and 120 h) in donkeys dosed orally with OTA (2500 μg/kg BW), *n* = 4.

As shown in Figure 2, after a single oral dose, the OTA in plasma was first detected at 5 min after administration, and increased gradually with time until 12 h, and then gradually decreased. The peak plasma concentration reached 10.34 ± 2.05 μg/mL (Table 2).

### 2.4. Recovery of OTA Eliminated in Urine and Feces

**Figure 3:**
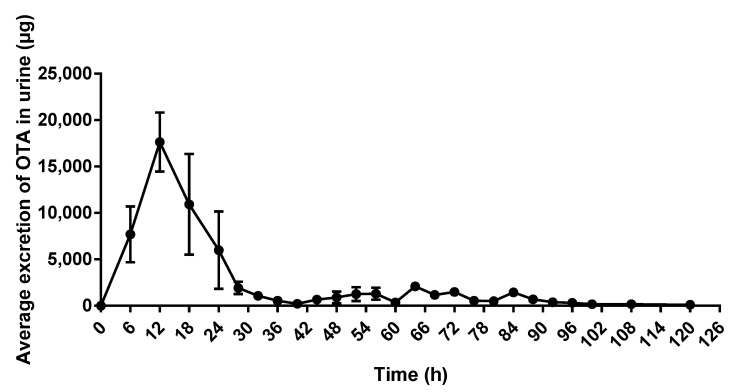
Average excretion of OTA in urine of donkeys treated with a single oral dose of 2500 μg OTA/kg BW, *n* = 4.

As shown in Figure 3, at 6 h after OTA administration, the elimination of OTA was detected, and rapidly increased between 6 h and 12 h. After 12 h, the amount of eliminated OTA began to decrease, and only low levels of OTA in urine were detected at 40 h.

**Figure 4:**
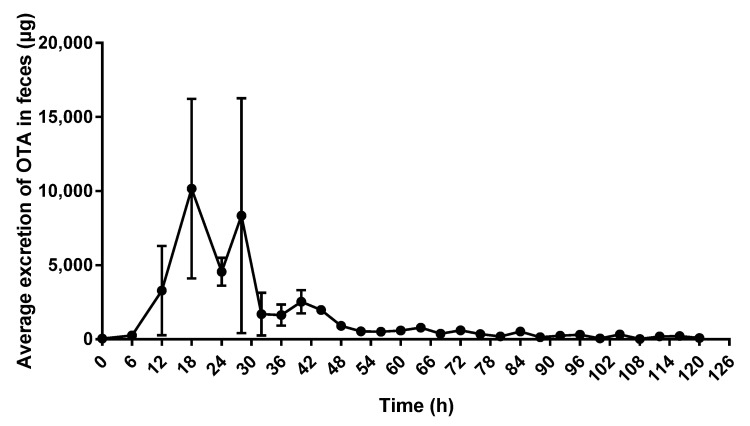
Average excretion of OTA in feces of donkeys treated with a single oral dose of 2500 μg OTA/kg BW, *n* = 4.

As shown in Figure 4, at 6 h after OTA administration, the elimination of OTA was low, and then rapidly increased until 18 h when it reached the peak concentration. After 18 h, the amount of eliminated OTA began to decrease, and only low levels of OTA in feces were detected at 52 h.

As shown in Table 4, the OTA intake for donkeys was 309.06 ± 5.21 mg. The total amount of OTA excreted through feces was 33.45 ± 15.63 mg, accounting for 10.85 ± 5.15% of the total intake. While the total amount of OTA excreted through urine was 37.69 ± 5.74 mg, accounting for 12.17 ± 1.73% of the total intake. The absorption rate was 89.15 ± 5.15%.

## 3. Discussion

Mycotoxicosis has been associated with several factors, including the toxin dose, time of consumption, the route of exposure, the sensitivity of the animal, and so on. Therefore, the metabolism of toxins varies in different species. Donkeys were used as the experimental subject because these animals are important for humans in providing meat and donkey-hide gelatin; however, there has been no studies about the effects of OTA in donkey after treatment with a single oral dose. In the present study, the toxicokinetics of a high dose of OTA for short periods of time were investigated.

The peak plasma concentration was positively correlated with the oral exposure dose. Studies have showed that the peak plasma concentration of male Wistar rats reached 35 μg/mL at a dose of 15 mg/kg [18], while the peak concentration of female Wistar rats was 0.39 μg/mL at a dose of 0.05 mg/kg BW [13]. With an oral exposure to male SD rats at a dose of 0.2 mg/kg BW, the peak plasma concentration reached 1.9 ± 0.1 μg/mL [19]. In the present study, the peak plasma concentration of donkeys reached 10.34 ± 2.05 μg/mL at a dose of 2.5 mg/kg BW. Consistent with other studies, the results indicate that the maximum level of OTA in donkey plasma were also associated with the oral exposure dose.

OTA can be combined with plasma proteins conducive to passive absorption. OTA has the characteristics of fast absorption and slow elimination when it enters the animal body, so it easily accumulates in the body [20]. In addition, the elimination half-life of OTA varies in different species, and may be related to the different degree of affinities for the plasma proteins [21]. High binding abilities of OTA to plasma proteins of various animals have been reported. Only 0.2% of the unbound toxin was in quail plasma, 0.1% in mouse plasma, and 0.08% in monkey plasma [13]. At the same time, the elimination half-life of carp, quail, mouse, pig, rat, monkey were 0.68, 6.7, 39, 72, 120, and 510 h, respectively, after oral administration [22]. In the present study, the elimination half-life for donkeys was 24.52 ± 2.48 h. The difference among different species may be because the plasma protein structure varies in different animals, resulting in different affinities, causing different absorption rates.

Fecal and urinary excretion ae two important pathways of toxin removal in all species. However, the high binding affinity of OTA to plasma albumin limits glomerular filtration and slows its excretion through the urine [16]. The relationship between the amount of OTA in urine with OTA intake is complex. It has been reported that there was no dose-dependent relationship between the amount of OTA intake and its excretion, and its rate of excretion in urine may depend on the plasma concentration. This means that when the plasma concentration was high, reabsorption decreased, leading to excretion increased [23]. In the present study, the maximum amount of OTA was excreted in urine at 12 h, and then began to gradually decrease, while the maximum peak of OTA in plasma also occurred at 12 h. This outcome is consistent with the conclusion reached by previous authors, which means that when the OTA concentration in plasma reaches its maximum, reabsorption reaches saturation and facilitates the excretion of OTA through urine. In addition, the metabolism of OTA needs to go through the enterohepatic circulation before excretion in feces [24]. Thus, the amount of OTA excreted through feces usually includes toxins that are not absorbed by the digestive tract, and toxins that are excreted through the bile into the intestines. It has been reported that after goats were given 0.5 mg OTA/kg BW in a single oral dose, the largest proportion (53%) of OTA was found in feces [25]. When calves were given 0.25 mg OTA/kg BW intravenously, 44.5% of administrated OTA was excreted through feces [26]. However, in the present study, only about 10% of the given dose of OTA was excreted through feces within 120 h, which is not consistent with other studies, in which a higher proportion of OTA was present in feces. This difference may be related to the species and dosage. We used a higher dose, compared to previous studies [25,26], which resulted in a longer retention time in vivo.

## 4. Conclusions

The present study revealed that after oral administration of 2.5 mg OTA/kg BW to donkeys, the OTA in the plasma reached a maximum at 12 h, and the elimination half-life was 24.52 ± 2.48 h. The maximum excretion of OTA in urine and feces occurred at 12 h and 18 h, respectively. The total excretion in both urine and feces was about 10% of the intake at 120 h. These results indicate the high absorption rate and slow elimination of OTA in donkeys.

## 5. Materials and Methods

### 5.1. Mycotoxins

OTA standards were purchased from Pribolab (Qingdao, China). The OTA used to treat the donkeys was produced by a toxigenic strain of *Aspergillus ochraceus* (CGMCC No. 3.4412) inoculated in corn for 3 weeks at 25 °C. The culture material was dried and ground to a fine powder. The culture material of *Aspergillus ochraceus* contained 640 mg/kg of OTA. Aflatoxin B1 (AFB1) and deoxynivalenol (DON) were not detected in the OTA material. The concentration of zearalenone (ZEN) and fumonisin B1 (FB1) in the culture material were 260.15 and 132.91 μg/kg, respectively.

### 5.2. Animals and Diet

Four healthy of 9-month-old Dezhou male donkeys were selected and placed in metabolism cages for collecting feces and urine during the experimental periods. After a five-day adaption period and fasting overnight, the body weights (BW) of donkeys were measured (123.6 ± 2.1 kg BW). The donkeys had free access to water and feed during the five-day experimental period. OTA, AFB1 and FB1 were not detected in concentrate feed or forage feed; the concentrations of ZEA and DON in the concentrate feed and forage feed were 139.48, 417.72 and 255.29, 408.79 μg/kg, respectively.

### 5.3. Toxin Administration

The culture material of *Aspergillus ochraceus* was administered orally in a single dose of 2500 μg OTA/kg BW. The material was dissolved in water and orally gavaged through an esophageal tube.

### 5.4. Blood, Plasma, Feces and Urine Collection

Blood was collected from the jugular vein in heparin anticoagulant tubes before administration and 5, 10, 15, 20, 30, 45 min, and 1, 1.5, 2, 3, 6, 9, 12, 24, 48, 72, 96 and 120 h after administration for analysis of OTA in plasma. In addition, samples of urine and feces were collected at 0, 6, 12, 18, 24, 28, 32, 36, 40, 44, 48, 52, 56, 60, 64, 68, 72, 76, 80, 84, 88, 92, 96, 100, 104, 108, 112, 116, 120 h for analysis of OTA.

### 5.5. Standard Solutions

A stock solution of 1 mg/mL OTA was prepared by dissolving 1 mg of OTA in 1 mL of methanol. The stock solution was diluted with methanol to prepare different concentrations of OTA working standard solutions (2, 10, 50, 100 and 500 μg/L, and 1, 5, 25, 50, 100 and 200 μg/mL). Blank plasma samples (90 μL) and blank urine samples (90 μL) were taken and 10 μL of different concentrations of working solutions were added to obtain eleven concentration levels of spiked samples in the range of 0.2–20,000 μg/L. In addition, 1 g of blank fecal samples was taken and 100 μL of different concentrations of working standard solution was added to obtain eleven concentration levels of spiked samples in the range of 0.2–20,000 μg/L. These samples were treated and detected according to the treatment and detection methods of plasma, urine and feces.

### 5.6. Sample Pretreatment

Pretreatment of plasma and urine samples was done according to the literature [19], as was the methods of fecal sampling [17]. The thawed plasma (100 μL) and urine samples (100 μL) were transferred into a 2 mL centrifuge tube, 300 μL of methanol added, vortexed for 1 min, and centrifuged at 12,000 rpm for 10 min. The supernatant was transferred into a 2 mL centrifuge tube, and evaporated in vials at 40 °C. Feces were dried and homogenized, and transferred 1.0 g into a 50 mL Erlenmeyer flask. A volume of 10 mL water was added and sonicated for 40 min. Then, the samples were transferred into 50 mL centrifuge tubes, adding 100 μL of 25% hydrochloric acid and 10 mL of chloroform, and then centrifuged at 12,000 rpm for 10 min. The extraction was repeated twice, and the liquid was collected and evaporated in a vial at 40 °C. Prior to HPLC analysis, the plasma and urine were reconstituted in 100 μL of methanol, feces samples were reconstituted in 1 mL of methanol, and all samples were vortexed for 1 min, and then filtered with a 0.22 μm filter for analysis by HPLC.

### 5.7. HPLC Method Validation

The method was validated according to linearity, sensitivity and recovery. These were studied individually for plasma, urine and feces. The calibration curves in three different blank matrices (plasma, urine and feces) were prepared at ten concentration levels in the range of 1–20,000 μg/L. Sensitivity was calculated by LOD and LOQ. At the lowest detection concentration calculation, signal to noise (S/N) of the LOD was ≥3 and S/N of the LOQ was ≥10. Recovery was obtained by comparing the peak area of five concentrations of OTA (1, 5, 100, 10,000 and 20,000 μg/L) of the spiked samples to the OTA peak area of the corresponding standard working solutions.

### 5.8. Detection of OTA in Plasma, Urine and Feces by HPLC

OTA was quantified using HPLC system equipped with a fluorescence monitor (RF-20A, Shimadzu, Kyoto, Japan) set at 333 nm excitation and 477 nm emission wavelengths. The injection volume was 20 μL, and the composition of mobile phase was acetonitrile, water and acetic acid (99:99:2, *v*/*v*/*v*) at a flow rate of 1.0 mL/min [4].

### 5.9. Statistical Analysis

Plasma OTA concentration-time data were calculated using the non-compartmental model method in WinNonlin 5.2.1 software to obtain the toxicokinetic parameters of OTA. At the same time, the average plasma OTA concentration was used as the OTA concentration-time curve. Average urine and feces of OTA excretion were used as the OTA excretion-time curve about urine and feces. Data were presented as mean ± SEM. The figures were drawn by using GraphPad Prism version 7.01 (GraphPad Software, Inc., San Diego, CA, USA).

## Figures and Tables

**Table 1:** Calibration curves of OTA in plasma, urine and feces.

Matrix	Slope	R^2^	Range (μg/L)/ (μg/kg)	Sensitivity (μg/L)/(μg/kg)	
				LOD	LOQ
Plasma	2483.4	0.9998	1–20,000	0.2	1.0
Urine	2063.1	0.9986	1–20,000	0.2	1.0
Feces	2128.2	0.9972	1–20,000	0.2	1.0

μg/L refers to the values of OTA in plasma and urine; μg/kg refers to the values of OTA in feces, *n* = 3 of each concentration.

**Table 2:** Recovery of OTA for plasma, urine and feces.

Spike Level (μg/L)/(μg/kg)	Recovery (%)		
	Plasma	Urine	Feces
1	88.81	99.10	97.55
5	75.34	89.15	84.24
100	96.94	86.80	76.60
10,000	88.34	77.93	72.56
20,000	98.06	80.62	85.00

*n* = 3 of each concentration.

**Table 3:** Toxicokinetic parameters of OTA in plasma of donkeys after a single oral dose of OTA.

Parameters	Value
Body weight (kg)	123.60 ± 2.09
OTA (μg·kg·BW^−1^)	2500
Tmax (h)	12.00 ± 0.00
Cmax (μg·mL^−1^)	10.34 ± 2.05
T_1/2_Elim (h)	24.52 ± 2.48
AUC (μg·mL^−1^·h)	656.20 ± 99.49
MRT (h)	48.58 ± 2.26
Cl (Cl/F) (L·kg·BW^−1^·h^−1^)	0.0041 ± 0.00
Vd (Vd/F) (L·kg·BW^−1^)	0.15 ± 0.036

Tmax: time of occurrence of maxima concentration of OTA in plasma. Cmax: concentration maxima of OTA in plasma,.T_1/2_Elim: terminal elimination half-life. AUC: area under plasma concentration-time curve. MRT: mean residence time. Cl: total plasma clearance. Vd: volume of distribution.

**Table 4:** Amount or percent of OTA in urine and feces in donkeys intoxicated with a single oral dose of OTA.

Parameters	Value
Body weight (kg)	123.60 ± 2.09
OTA (mg·kg·BW^−1^)	2.50
OTA intake (mg)	309.06 ± 5.21
OTA excretion through feces (mg)	33.45 ± 15.63
OTA excretion through feces (%)	10.85 ± 5.15
OTA excretion through urine (mg)	37.69 ± 5.74
OTA excretion through urine (%)	12.17 ± 1.73
Absorption rate (%)	89.15 ± 5.15

OTA excretion through feces (%) = OTA excretion through feces (mg)/OTA intake (mg) × 100. OTA excretion through urine (%) = OTA excretion through urine (mg)/OTA intake (mg) × 100. Absorption rate (%) = (OTA intake (mg) − OTA excretion through feces (mg))/OTA intake (mg) × 100.

## Data Availability

The data presented in this study are available on request from the corresponding author.
